# Artificial intelligence applied in cardiovascular disease: a bibliometric and visual analysis

**DOI:** 10.3389/fcvm.2024.1323918

**Published:** 2024-02-16

**Authors:** Jirong Zhang, Jimei Zhang, Juan Jin, Xicheng Jiang, Linlin Yang, Shiqi Fan, Qiao Zhang, Ming Chi

**Affiliations:** ^1^ Graduate School, Heilongjiang University of Chinese Medicine, Harbin, Heilongjiang, China; ^2^ College of Public Health, The University of Sydney, NSW, Sydney, Australia; ^3^ The First Department of Cardiovascular, First Affiliated Hospital of Heilongjiang University of Chinese Medicine, Harbin, HL, China; ^4^ College of basic medicine, Heilongjiang University of Chinese Medicine, Harbin, HL, China; ^5^ Cardiovascular Disease Branch, Dalian Second People's Hospital, Dalian, LN, China; ^6^ Harbin hospital of traditional Chinese medicine, Harbin, HL, China; ^7^ School of Pharmacy, Harbin University of Commerce, Harbin, HL, China

**Keywords:** cardiovascular disease, artificial intelligence, late gadolinium enhancement, Left Ventricle Ejection Fraction (LVEF), bibliometric

## Abstract

**Background:**

With the rapid development of technology, artificial intelligence (AI) has been widely used in the diagnosis and prognosis prediction of a variety of diseases, including cardiovascular disease. Facts have proved that AI has broad application prospects in rapid and accurate diagnosis.

**Objective:**

This study mainly summarizes the research on the application of AI in the field of cardiovascular disease through bibliometric analysis and explores possible future research hotpots.

**Methods:**

The articles and reviews regarding application of AI in cardiovascular disease between 2000 and 2023 were selected from Web of Science Core Collection on 30 December 2023. Microsoft Excel 2019 was applied to analyze the targeted variables. VOSviewer (version 1.6.16), Citespace (version 6.2.R2), and a widely used online bibliometric platform were used to conduct co-authorship, co-citation, and co-occurrence analysis of countries, institutions, authors, references, and keywords in this field.

**Results:**

A total of 4,611 articles were selected in this study. AI-related research on cardiovascular disease increased exponentially in recent years, of which the USA was the most productive country with 1,360 publications, and had close cooperation with many countries. The most productive institutions and researchers were the Cedar sinai medical center and Acharya, Ur. However, the cooperation among most institutions or researchers was not close even if the high research outputs. *Circulation* is the journal with the largest number of publications in this field. The most important keywords are “classification”, “diagnosis”, and “risk”. Meanwhile, the current research hotpots were “late gadolinium enhancement” and “carotid ultrasound”.

**Conclusions:**

AI has broad application prospects in cardiovascular disease, and a growing number of scholars are devoted to AI-related research on cardiovascular disease. Cardiovascular imaging techniques and the selection of appropriate algorithms represent the most extensively studied areas, and a considerable boost in these areas is predicted in the coming years.

## 1 Introduction

Cardiovascular Disease (CVD) stands as a major health concern worldwide, serving as a prime contributor to the global mortality rate. As per the World Health Organization's 2017 report, CVDs have been implicated in an alarming estimate of 17.9 million deaths (1). The term “CVD” includes a plethora of medical conditions such as ischemic heart disease, arrhythmias, hypertension, acute coronary syndrome, and myocardial infarction. Tragically, despite various global health initiatives and advancements in medical technology, the worldwide burden of CVD has continued to witness a steady rise over the past two decades (2). Furthermore, despite a notable amplification in the global life expectancy by 7.4 years over the past two decades and an enhancement in CVD survival rates by 10.3% between 2007 and 2017, mortality rates stemming from CVD persist at high levels - in some age groups and population demographics, this percentage peaks at 13.7% (3). These sobering statistics spotlight the urgent and undeniable need to bolster the drive for more thorough and multifaceted research into CVD prevention and treatment measures.

The rapid evolution of information and communication technology has precipitated a major shift in disease treatment methodologies. In recent years, personalized medicine, which is also referred to as precision medicine, has gained significant popularity and has opened new avenues for clinical scenarios (4). This paradigm shift is remarkably pronounced in the cardiovascular arena, where the intricate techniques utilized and the substantial volume of data generated present formidable challenges for medical practitioners (5). However, it is worth noting that an unavoidable problem brought about by personalized medicine on a global scale is the difficulty in maintaining quality control by health administration departments (6, 7). In response to this complex landscape, there is a compelling drive toward adopting streamlined, precise, and automated systems within the medical community. For example, developing and improving tools and algorithms for integrating and analyzing biomedical data in a system (8). These systems can significantly bolster the prevention, diagnosis, and treatment of CVDs (9). Concurrently, this technological revolution can potentially curtail medical expenses and critically reduce the fatality rate associated with CVDs, making it an essential endeavor in public health.

In fact, the potential application of Artificial Intelligence (AI) is exciting. Since its conception in the 1950s by McCarthy et al., AI has progressively developed as a rapidly proliferating multidisciplinary sector (10). Incorporating diverse fields such as computer science, statistics, psychology, neuroscience, material science, mechanical engineering, and computer hardware design, AI strives to devise algorithms that emulate human intuition, decision-making capabilities, and object recognition. Owing to the robust potential of its advanced algorithms and learning abilities, AI's application in the medical realm has expanded. It now includes disease diagnosis, prognosis prediction, drug research, and genomic data analysis, amid other areas. Using AI models like logistic regression (LogR) (11), support vector machine (SVM) (12), artificial neural network (ANN) (13), and convolutional neural network (CNN) (14) has yielded significant improvements in CVD detection, diagnosis, and risk prediction (15). While substantial parts of this research are still in their preliminary stages, there is already evidence that underscores the impressive potential of AI in interacting with CVDs (16).

A prevalent method for systematically reviewing and presenting AI-based CVD research is Bibliometrics. Notably, its utility shines when surveying the cutting edge of current investigations (17). This method casts a spotlight on the achievements of researchers, institutions, and emerging scholars, offering an in-depth perspective that often surpasses traditional systematic reviews and clinical observations (18–20). By evaluating global scientific articles and the latest frontier research, bibliometrics allows for a comprehensive understanding of scientific publications and provides a graphical representation of their trends. Pursuing this method can enhance comprehension of the state-of-the-art research progress, which aligns with the core objective of this review paper focusing on the application of AI in the cardiovascular field.

Therefore, we employ the bibliometric analysis method to comprehensively examine the literature on AI in CVDs, from WOS from 1 January 2000 to 30 December 2023. Our approach will focus on three primary aspects: (i) We aim to quantify the data on AI in CVDs research to identify the most productive countries, institutions, journals, authors, and co-authors. (ii) We plan to display the foundational knowledge and development trends in the AI in CVDs field through co-cited reference analysis. (iii) Lastly, and most importantly, to assist scholars in clarifying their research direction, we will identify the current focal points or hotpots in AI research concerning CVDs.

## 2 Materials and methods

### 2.1 Data collection and search strategy

**Figure 1:**
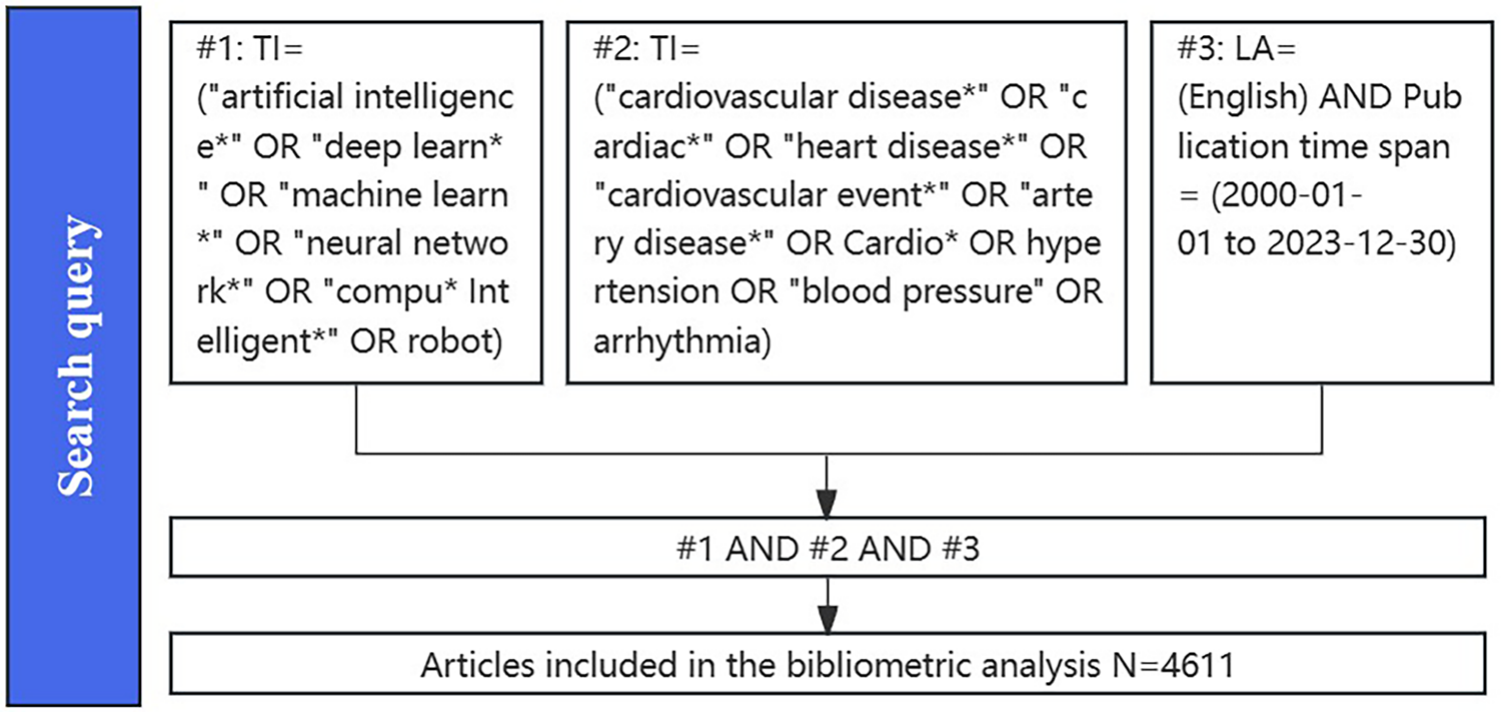
Flow diagram of screening progress related to AI in CVDs.

We searched for relevant articles in Clarivate Analytics' Web of Science Core Collection (WoSCC), which is among the most comprehensive and influential databases in interdisciplinary fields. It encompasses over 9,000 research journals and offers various bibliometric indicators (e.g., titles, keywords, country/region, institutions, and categories), making it widely utilized as a data source for bibliometric research. All publications were retrieved and downloaded from the WoSCC database on 30 December 2023. Three independent researchers (Linlin Yang, Ming Chi and Shiqi Fan) conducted the literature search to ensure the reliability and authenticity of the results. The search strategy was developed based on previous research, and the detailed approach is as follows: TI = (“cardiovascular disease*” OR “cardiac*” OR “heart disease*” OR “cardiovascular event*” OR “artery disease*” OR Cardio* OR hypertension OR “blood pressure” OR arrhythmia) AND TI = (“artificial intelligence*” OR “deep learn*” OR “machine learn*” OR “neural network*” OR “compu* Intelligent*” OR robot) AND LA = (English) AND Publication time span = (1 January 2000 to 30 December 2023). We followed the search strategy and screening process, resulting in a total of 4,656 documents. Details of the retrieval procedure are provided in Figure 1. Finally, all included documents are exported as “complete records and references.” All records, including titles, authors, abstracts, keywords, etc., are imported into CiteSpace for summarizing and visualizing scientific literature.

### 2.2 Data analysis and visualization

The WoSCC literature analysis report was utilized to evaluate publication characteristics, including output in terms of the number of annual publications, journals, authors, citation frequency, impact factor (IF), and H-index. The H-index was employed to assess the scientific impact of the author or country. Excel was used for analyzing publication trends, and the growth of publications in the following year was estimated using the polynomial model. For bibliometric analysis and visualization, we used Citespace 6.2.R2, VOSviewer 1.6.19, and the Online Analysis Platform of Literature Metrology (https://bibliometric.com/).

Citespace, a widely used visualization software in bibliometrics, is commonly employed to detect hot spots, potential trends, and evolutionary progress in research fields (21–23). Using CiteSpace, we assessed keyword timelines and citation bursts of keywords or references. CiteSpace parameters were configured as follows: (1) Time slice from 2003 to 2023, with each slice representing one year; (2) Single node type selection at a time; (3) Selection criteria defined as Top *N* = 25; (4) Pruning performed using the pathfinder and pruning sliced networks. Additionally, this study employed VOSviewer for creating, visualizing, and exploring knowledge maps based on network data. We primarily used VOSviewer to conduct author-keyword co-occurrence analysis, co-authorship analysis of countries/regions, authors, institutions, and co-citation analysis of journals or references. A larger link width between nodes indicated a stronger degree of cooperation, while a larger node size denoted a greater number of reflections. Nodes of the same color belonged to the same cluster. The Online Analysis Platform of Literature Metrology was employed for conducting country/region co-authorship and publication analyses.

### 2.3 Research ethics

The data sources for our study were obtained from public databases; therefore, no ethical permission was required for this research.

## 3 Result

### 3.1 General information

**Figure 2:**
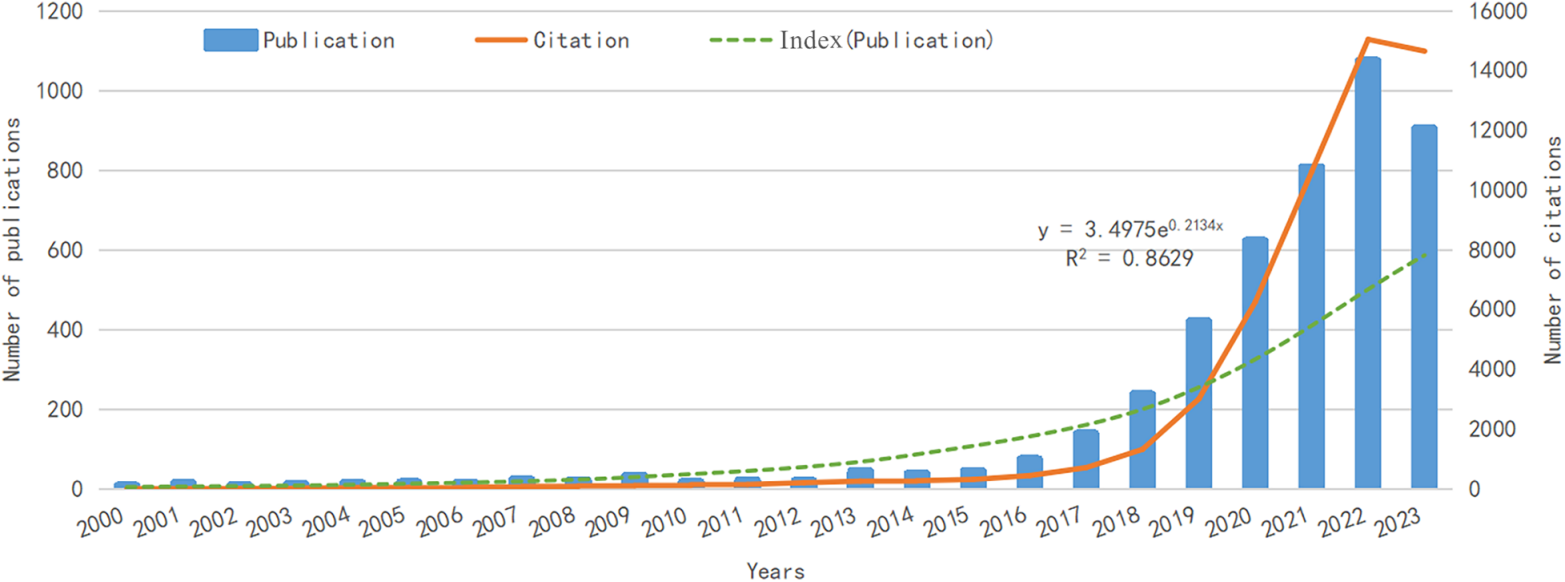
Global trend of publications on AI-based CVDs pathology research over the past 23 years.

Following our data search strategy, we gathered 4,611 articles from the WOS database spanning the last 23 years (Figure 1). The trend of annual publications is illustrated in Figure 2. Analysis reveals that, (i) in the initial 10 years (2000–2010), fewer than 50 articles on AI in CVD research were published annually; (ii) in the subsequent 5 years (2011–2015), the annual publication rate experienced smooth fluctuations; (iii) in the recent 8 years (2016–2023), there has been a rapid increase in the number of published articles.

**Figure 3:**
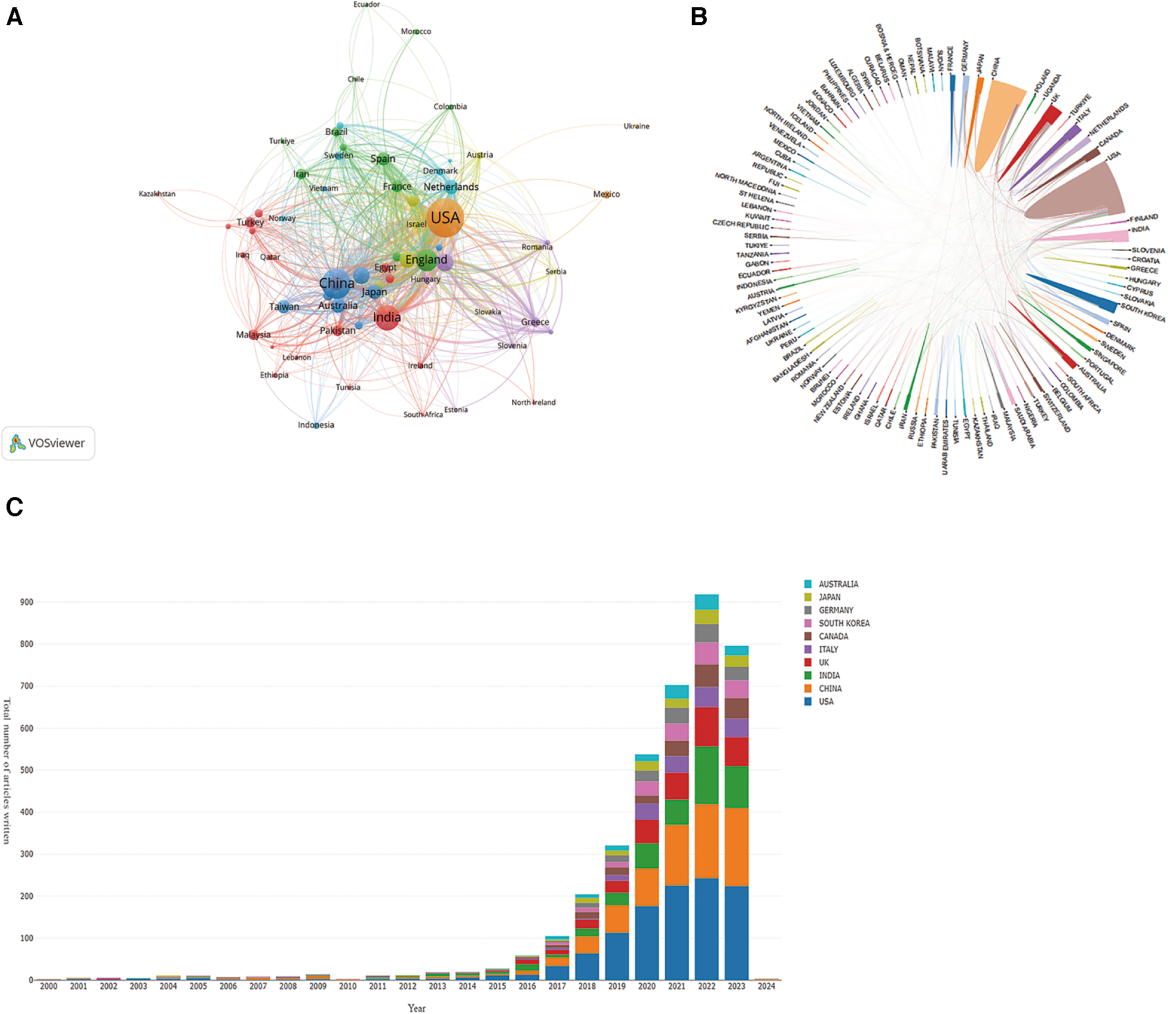
Visual map of countries/region. (**A**) The countries/regions citation overlay visualization map generated by using VOS viewer. (**B**) Collaboration network analysis of countries/regions. (**C**) The distribution trend of the top 10 countriesregions by year.

Publications on AI in CVDs originated from 113 countries. Using VOSviewer with a threshold of a minimum of 5 documents per country, we identified 70 nodes and 7 clusters (Figure 3A). Nearly 89.4% of total articles came from the top 10 countries (Table 1). The USA led with 1,360 articles (29.4%), surpassing other leading countries such as England with 411 articles (8.9%) and Canada with 237 articles (5.1%). The USA also recorded the highest H-index value of 67, signaling its leadership. In terms of centrality, the USA is first, followed by England and China. Co-authorship country analysis in Figures 3A,B highlights the USA as one of the most collaborative countries, particularly with Canada and England. Importantly, our analyses reveal rapid progression in Chinese research outputs over the past 7 years (Figure 3C).

**Table 1:** The top 10 productive countires/regions in AI-based CVDs research (sorted by TLS value; TLS = total link strength, the total strength of the links of an item with other items).

Rank	Country	TLS	H-index	Counts	Centrality
1	USA	1,020	67	1,360	0.28
2	ENGLAND	581	41	411	0.30
3	CANADA	411	28	237	0.02
4	ITALY	376	31	255	0.07
5	GERMANY	374	28	208	0.08
6	INDIA	322	33	484	0.01
7	CHINA	284	40	716	0.1
8	NETHERLANDS	237	26	160	0.14
9	SAUDI ARABIA	237	19	134	0.07
10	AUSTRIA	216	27	157	0.11

**Figure 4:**
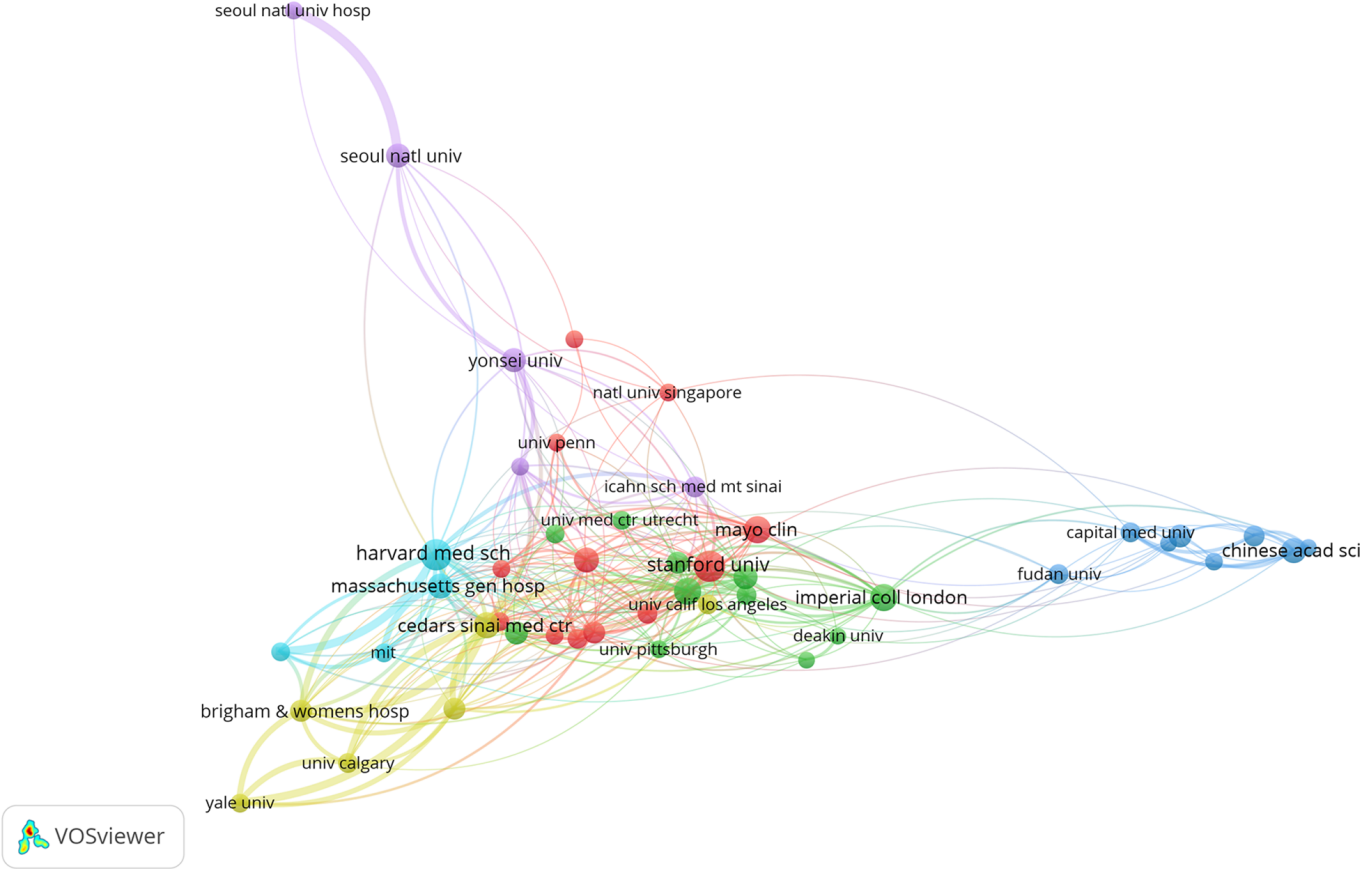
The network of institution.

To explore core institutions and cooperation relationships in AI in cardiovascular research, we generated a network of co-authors' institutions. The font size of an organization is proportional to the number of publications. From Figure 4, we can identify the institutions that contribute the most papers and/or occupy crucial positions in the largest subnetwork. Publications were published in 4,973 different institutions. Table 2 lists the top 10 most productive institutions. The Cedar Sinai Medical Center published the most papers (*n* = 1,249, 27%), followed by Harvard Medical School (*n* = 862, 18.7%) and Brigham Women's Hospital (*n* = 538, 11.7%). Using VOSviewer with a threshold of a minimum of 20 per institution, we identified 46 nodes and 6 clusters. The connection and thickness between nodes represent the cooperation relationship and frequency between two institutions.

**Table 2:** The top 10 organizations in AI-based CVDs research (sorted by TLS value; TLS = total link strength, the total strength of the links of an item with other items).

Number	Organizations	TLS	Documents	Citations	H-index
1	Cedar sinai medical center	93	51	1,249	22
2	Harvard medical school	84	72	862	20
3	Brigham women s hospital	74	35	538	20
4	Massachusetts general hospital	56	45	328	17
5	Columbia university	55	34	654	14
6	Stanford university	53	71	3,682	24
7	University of Oxford	45	56	978	17
8	Yale university	45	26	301	11
9	Imperial college London	44	55	1,751	19
10	Cleveland clinic foundation	41	27	929	16

**Figure 5:**
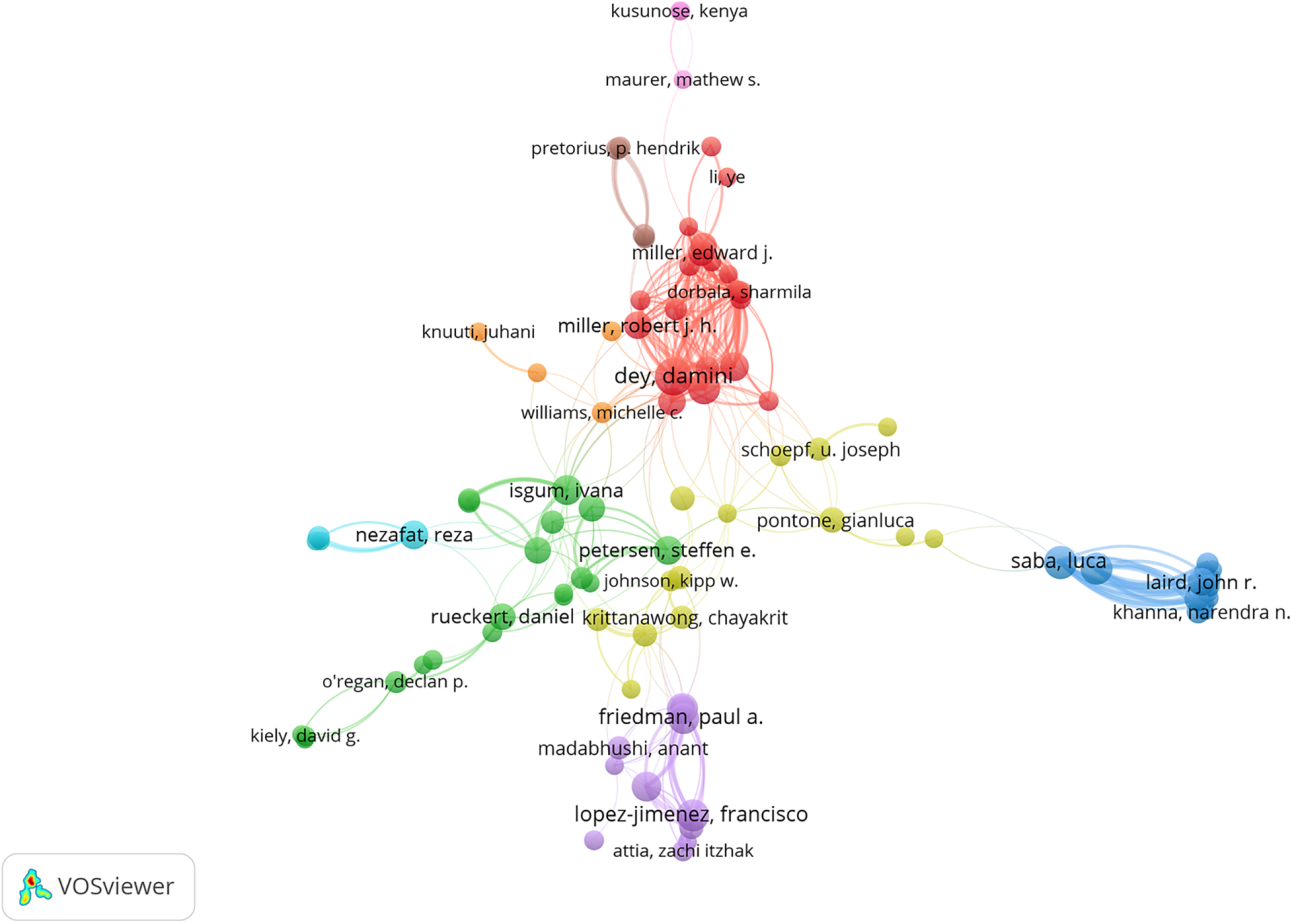
A visual authors co-operation map for VOSviwer network.

In total, 19,646 co-authors contribute to AI in CVDs field. The cooperation map (Figure 5) indicates that the scale of collaboration among authors is relatively light, suggesting the need for increased overall connections between researchers in this field.

Co-cited authors are those whose papers are cited by one or more other publications simultaneously. Table 3(A) is the ranking of authors' publications in this field. The H index in the table is a standard for measuring the quantity and level of academic output (24). Most authors show a positive correlation with H-index. Among the top 10 co-cited authors, there were more than 1,000 citations, as shown in Table 3(B). Acharya, Ur (*n* = 511) was the most frequently co-cited author, followed by Saba, L, f (*n* = 151), and Molinari, F (*n* = 93). Table 3 presents the top 5 authors in terms of publications, with Dey, Damini, ranking first with 39 articles.

**Table 3(A):** Top 10 authors in AI-based CVDs research (sorted by article value).

Rank	Author	H-index	Article	Country
1	Damini Dey	53	39	ENGLAND
2	Slomka, Piotr J	66	36	USA
3	Noseworthy, Peter A.	55	35	USA
4	Attia, Zachi Itzhak	20	32	USA
5	Saba, Luca32	44	12	ITALY

**Table 3(B):** Top 10 authors TLS (sorted by TLS value; TLS = total link strength, the total strength of the links of an item with other items).

Rank	Author	Citiations	TLS	Rank	Author	Citations	TLS
1	Acharya, Ur	511	11,637	6	Goldberger, Al	230	2,991
2	Saba, L	151	7,448	7	Breiman, L	316	2,990
3	Molinari, F	93	4,398	8	Krittanawong, C	168	2,945
4	Suri, Js	75	4,306	9	Jamthikar, Ad	54	2,896
5	Khanna, Nn	62	3,135	10	Martis, R	155	2,893

### 3.2 Hotspot evolution analysis

#### 3.2.1 Journals and cited journals

Since 2000, a total of 18,617 academic journals published articles related to the field of AI research concerning CVDs. In terms of the number of co-citations (Table 4), the top three were *Circulation* (IF = 37.83), *J Am Coll Cardiol* (IF = 24.4), and *Journal of the European Heart Journal* (IF = 39.3). The top 5 journals published 12.6% (*n* = 581) of the papers, as shown in Table 5. Among these journals, the average IF of the top 5 is 21.86. And the *European Heart Journal* (*n* = 117) had the highest IF among journals. In terms of the number of publications, the top three were *Circulation* (IF = 37.8), *Journal of the American College of Cardiology* (IF = 24), and *European Heart Journal* (IF = 39.3).

**Table 4:** Top 10 most productive journals.

Rank	Journal	Count	Country	IF (2022)	JCR (2022)
1	CIRCULATION	207	USA	37.8	Q1
2	JOURNAL OF THE AMERICAN COLLEGE OF CARDIOLOGY	147	USA	24.4	Q1
3	EUROPEAN HEART JOURNAL	117	ENGLAND	24	Q1
4	FRONTIERS IN CARDIOVASCULAR MEDICINE	107	SWITZERLAND	3.6	Q3
5	SCIENTIFIC REPORTS	80	GERMANY	4.6	Q2

**Table 5:** Top 10 most cited journals.

Rank	Co-cited Journal	Count	Country	IF (2022)	JCR (2022)
1	CIRCULATION	1,713	USA	37.8	Q1
2	J AM COLL CARDIOL	1,270	USA	24.4	Q1
3	EUR HEART J	1,015	ENGLAND	39.3	Q1
4	PLOS ONE	954	USA	3.7	Q2
5	SCI REP-UK	846	ENGLAND	4.6	Q1

#### 3.2.2 Analysis of co-cited references

7 papers with numbers greater than 100 are listed in Table 6, sorted by citation counts reports. The most cited article was the review published by Hannun, Awni Y. in 2019, with a total of 153 times, followed by a review published by Johnson KW (2018), 126 times. Attia ZI. (2019), 117 times. And a review published by Motwani M (2017), 109 times respectively.

**Table 6:** Top 7 articles according to number of citations.

	Title	First author	Journal	IF (2022)	JCR (2022)	Years	Citation
1	Cardiologist-level arrhythmia detection and classification in ambulatory electrocardiograms using a deep neural network	Hannun, Awni Y.	Nature Medicine	82.9	Q1	2019	171
2	Artificial Intelligence in Cardiology	Johnson KW	J AM COLL CARDIOL	24.4	Q1	2018	126
3	Screening for cardiac contractile dysfunction using an artificial intelligence–enabled electrocardiogram	Attia ZI	NAT MED	82.9	Q1	2019	117
4	Machine learning for prediction of all-cause mortality in patients with suspected coronary artery disease: a 5-year multicentre prospective registry analysis	Motwani, M.	European Heart Journal	39.3	Q1	2017	109
5	Automated cardiovascular magnetic resonance image analysis with fully convolutional networks	Wenjia Ba	Journal of Cardiovascular Magnetic Resonance	6.4	Q1	2018	105
6	ImageNet classification with deep convolutional neural networks	Krizhevsky, Alex	Communications of the ACM	22.7	Q1	2017	102
7	Deep Learning Techniques for Automatic MRI Cardiac Multi-Structures Segmentation and Diagnosis: Is the Problem Solved?	Bernard, O.	Iee Transaction Medicl Imaging	10.6	Q1	2018	102

**Figure 6:**
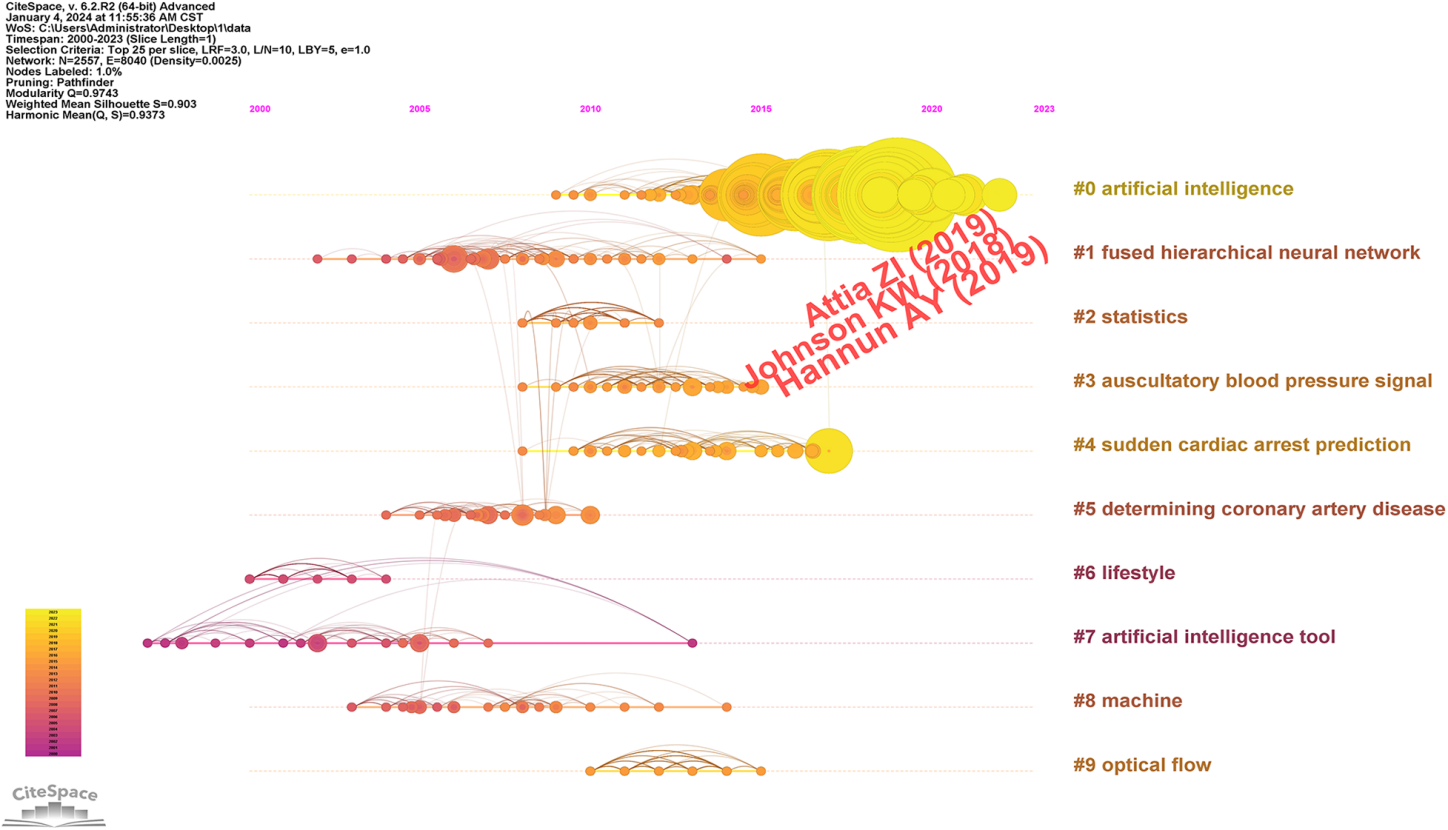
Co-cited references timeline view.

**Figure 7:**
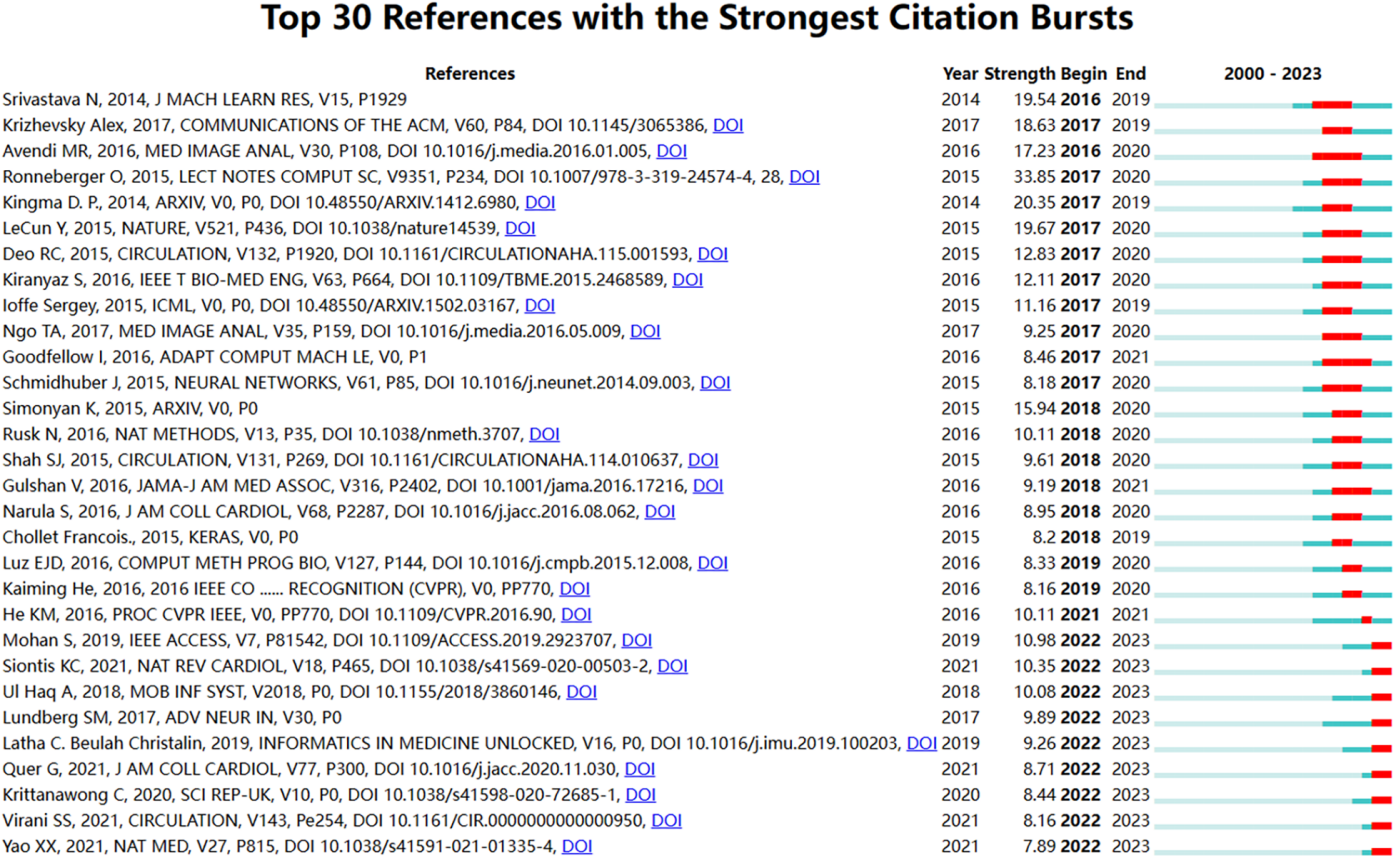
Top 30 references with the strongest citation bursts on AI-based CVDs research.

The analysis results from Figure 6 indicate that the Modularity Q was 0.9743, and the mean Silhouette S was also notably high at 0.903, demonstrating the excellent clustering effect and impressive network homogeneity. The timeline view of the co-citation references presents the evolution of research hotpots over time. According to the clustering results, the research can be divided into ten clusters. Currently, the latest research hotpots are “artificial intelligence” (#0), “cardio electrophysiology” (#4), “heart disease prediction” (#5) and “using deep learning” (#8), conveying that an increasing number of researchers are focusing on the application of deep learning and precision medicine in the treatment of CVDs. Figure 7 lists the top 30 references with the most significant citation bursts. Most of these pivotal articles continue to be cited frequently. One notable entry among these is the paper published in 2021, which is the most recent citation burst strength and continues up to the present (25).

#### 3.2.3 Keyword co-occurrence and burst detection

The keyword serves as the cornerstone of an article. Through the process of keyword co-occurrence analysis, the evolving trends of research topics can be unearthed over time, thereby enabling the tracking of scientific progress.

**Figure 8:**
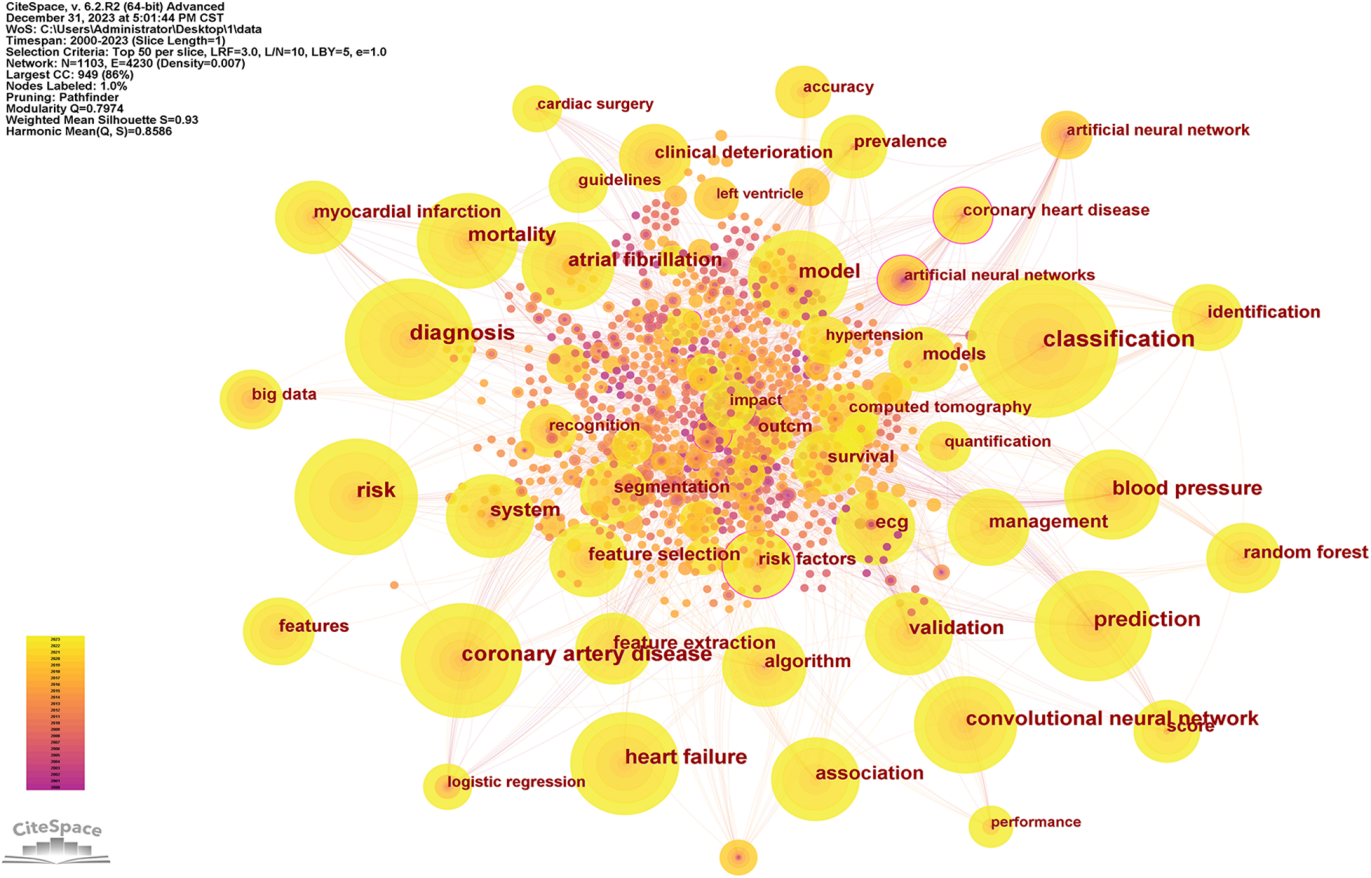
The network of keywords.

**Figure 9:**
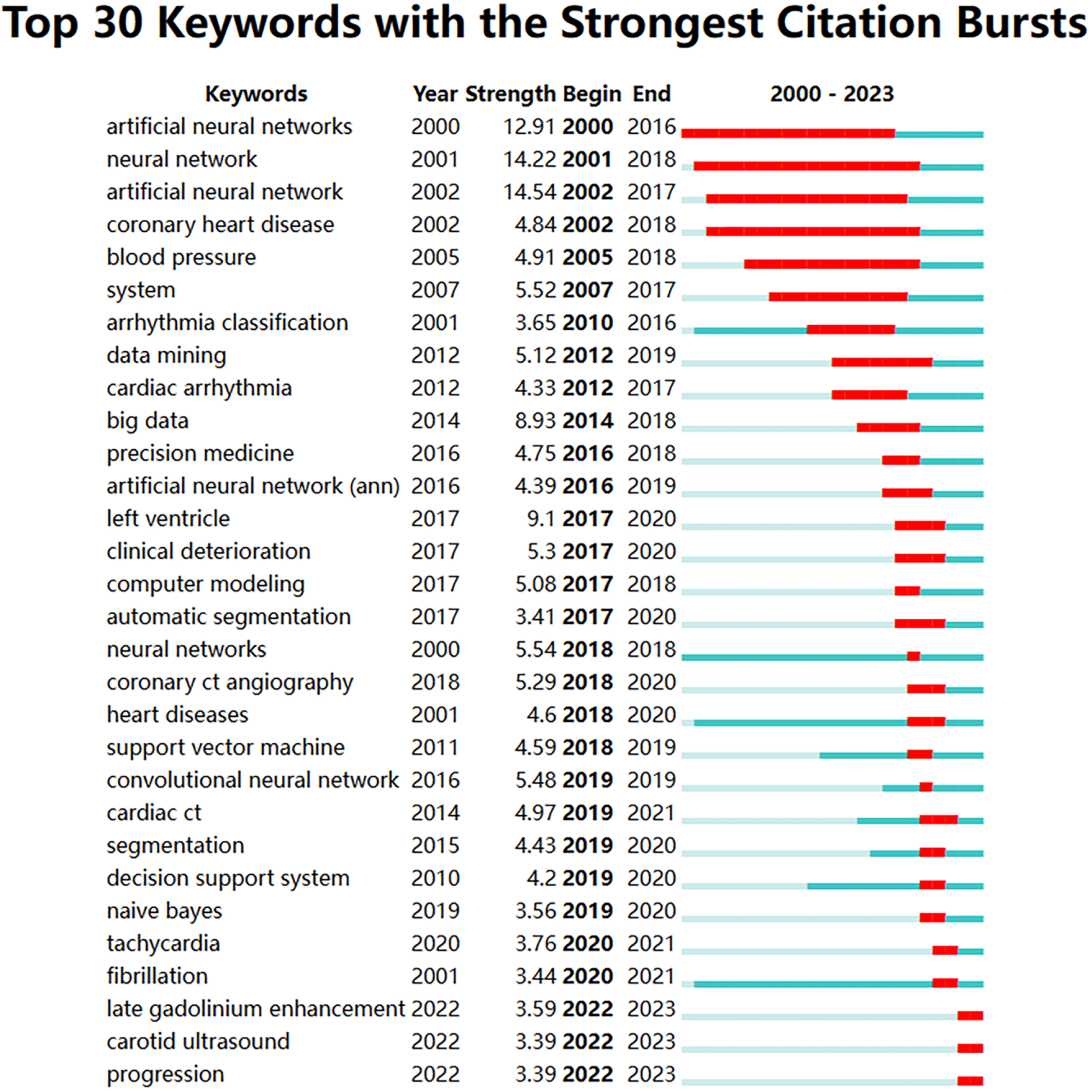
Top 30 keywords with the strongest citation bursts.

**Figure 10:**
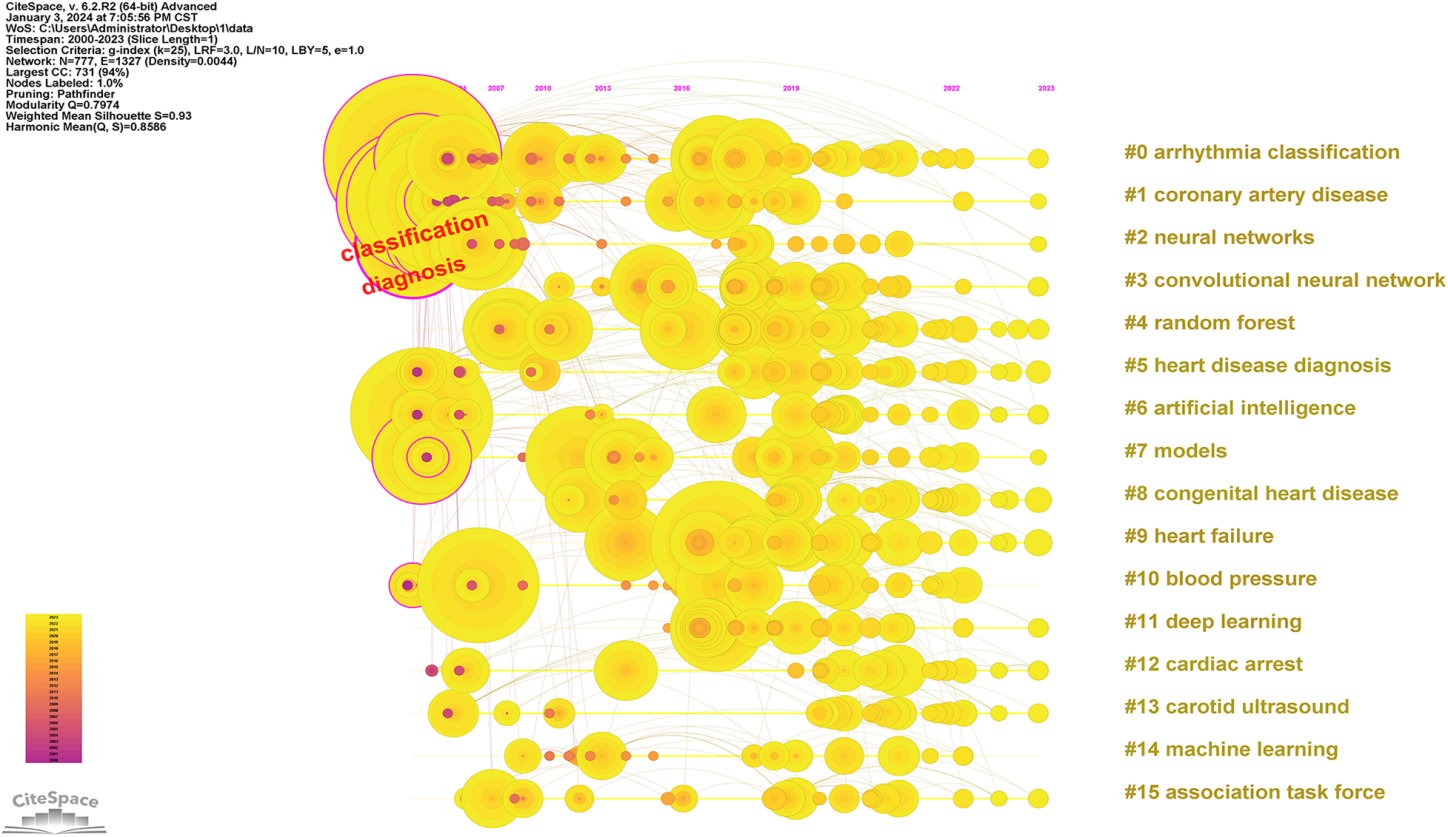
Timeline view to AI in CVDs.

After resetting Citespace to one year per slice and setting the pruning options to “Pathfinder,” “Pruning sliced networks,” and “Pruning the merged network,” with a top *N* of 50, the process yielded 4,015 qualified records. These records were organized based on the co-occurrence frequency of the keywords. Figure 8 analysis results, the Modularity Q was 0.7974, and the mean Silhouette S was also as high as 0.93 The terms in Figure 8 were presented in Table 7 with their corresponding number of occurrences. A centrality value exceeding 0.1 indicates that the node is central in the field and carries significant influence in the research. According to the centrality ranking, the top three keywords are “artificial neural networks” “neural networks” and “classification”. After excluding the search term of this study, we got three significant words which are “classification” “diagnosis” and “acute myocardial infarction”. Presently, these constitute the primary subjects of focus in our research field. Figures 9 and 10 utilize a timeline perspective along with a keyword burst tool, effectively demonstrating the cutting-edge of scholarly research.

**Table 7:** Top 50 keywords in the field of AI for cardiovascular.

Rank	Keywords	Occurrences	Centrality	Rank	Keywords	Occurrences	Centrality
1	machine learning	1,086	0	26	feature extraction	101	0.05
2	deep learning	721	0	27	algorithm	100	0.03
3	artificial intelligence	560	0.02	28	feature selection	91	0.01
4	classification	347	0.05	29	myocardial infarction	90	0.04
5	diagnosis	261	0.1	30	random forest	81	0.03
6	risk	235	0.03	31	risk factors	80	0.12
7	coronary artery disease	234	0.08	32	features	78	0
8	prediction	216	0.07	33	identification	77	0.08
9	cardiovascular disease	214	0.06	34	clinical deterioration	77	0
10	heart disease	209	0.07	35	cardiovascular diseases	76	0.01
11	heart failure	178	0	36	outcm	74	0.01
12	convolutional neural network	163	0.01	37	models	73	0
13	model	163	0.09	38	segmentation	71	0
14	mortality	163	0.08	39	prevalence	71	0.07
15	neural networks	151	0.11	40	survival	70	0
16	neural network	150	0.05	41	health	69	0
17	blood pressure	149	0.07	42	score	68	0.01
18	disease	142	0.02	43	big data	61	0.02
19	atrial fibrillation	135	0.05	44	coronary heart disease	58	0.1
20	system	133	0.02	45	computed tomography	57	0.01
21	validation	129	0.07	46	guidelines	54	0.01
22	association	123	0.03	47	recognition	53	0.05
23	heart	120	0.07	48	artificial neural network	48	0.07
24	ecg	112	0.04	49	accuracy	47	0
25	management	109	0.03	50	quantification	45	0.03

## 4 Discussion

### 4.1 General information

In the current study, a comprehensive review of articles published over the last 23 years (2000–2023), focusing on AI in CVDs, was conducted using the WOSCC databases. A total of 4,656 papers were found. After applying the screening criteria and omitting documents that met exclusive criteria, this bibliometric study included 4,611 papers. These were authored by 908 contributors from 630 organizations in 113 countries/regions and were published in 1,731 journals, with 1,167 co-cited references included.

The annual scientific production of AI in CVDs has shown a significant upward trend, particularly over the last eight years. This period, characterized by sharp growth, accounted for 98.6% of publications, with a total of 4,268. These findings highlight the evident interdisciplinary integration characteristics of AI in CVDs research. The USA holds a leading position in the field of AI in CVDs. Notably, despite a late start, China has become the second most productive nation in paper publication globally, establishing close collaboration with the USA, the field's epicenter. It is anticipated that more countries and researchers will partake in AI research concerning CVDs studies in the future. Among the top 10 most productive institutions, all originated from merely two different countries, with the USA housing 80% of them, and the other 20% based in the UK. The Cedar Sinai Medical Center is the most prolific institution, showcasing the fact that machine learning algorithms have already permeated clinical cardiology.

The centrality of Damini Dey and Slomka, Piotr J places them as the top two authors in this field, indicating their influential status. Reading their publications would be advantageous to gaining an understanding of the knowledge structure in this field. The journal of *Circulation* boasts a significantly higher citation count compared to other journals. According to the 2022 JCR, among the top five journals, three are categorized in Q1, indicating the high quality of their papers. Furthermore, the Journal of the *IEEE ACCESS* and the *LECTURE NOTES IN COMPUTER SCIENCE* are also high-yielding publications. *FRONTIERS IN CARDIOVASCULAR MEDICINE* and *SCIENTIFIC REPORTS* likewise demonstrate the potential to publish more high-quality articles in the future, thereby enhancing their academic stature and impact factor.

### 4.2 Research hotpots

The categorization of artificial intelligence systems in medical applications demonstrates the potential to aid clinicians and researchers in processing large volumes of medical data, including medical images and records. This can enable a more efficient diagnosis and analysis process. Emerging research illustrates that AI-informed health management systems can perform a variety of autonomous or semi-autonomous tasks, such as cardiology diagnosis (26), treatment (27), self-monitoring (28), and disease risk assessment (9). As highlighted by Hannun et al., deep neural networks, when used in the analysis of 12-lead electrocardiograms, resulted in a better classification accuracy, compared to cardiologists’ conventional analysis methods (29). Another study by Gupta et al. showcased the successful use of a ConvNetQuake neural network model to accurately diagnose Acute Myocardial Infarction (AMI) from ECGs, achieving an impressive accuracy rate of 99.43% (30).

Bibliometric studies often utilize citation and co-citation analyses of references to determine notable literature, evaluate research progression, and predict potential future research directions. Articles with a significant number of citations typically illustrate high-quality research marked by substantial innovation and remarkable influence within their respective fields. An analysis of the most-cited studies underscores the impact of this particular field (31).

Notably, an article in 2019 by Awni Y. Hannun titled “Investigating Deep Learning in Ambulatory Electrocardiogram Analysis”, published in Nature Medicine, became the most-cited publication in this field with 153 citations (IF = 82.9) (29). The paper focused on the development of a deep neural network (DNN) that achieved an average area under the Receiver Operating Characteristic curve (ROC) of 0.97. This DNN's average F1 score (0.837) was found to be higher than that of cardiologists (0.780), thereby demonstrating that an end-to-end deep learning approach can match, or even exceed, the diagnostic performance of cardiologists in classifying a wide range of arrhythmias from single-lead electrocardiograms. A 2017 article by M. Motwani and team focused on the feasibility and accuracy of machine learning (ML) to predict five-year all-cause mortality (ACM) in patients with suspected Coronary CT Angiography (CCTA), comparing existing clinical or CCTA metrics (32).

Equally influential, Johnson KW et al. published a review in 2018 which discussed the current research status and application of AI technology in cardiovascular medicine at that time, and comprehensively and systematically summarized that in addition to analyzing data to predict the risk and development trend of heart disease, AI can also carry out the diagnosis of heart disease, provide more accurate results and recommendations, and assist doctors to improve the accuracy and safety of surgery. It also expresses the importance of accurate algorithms (e.g., CNNs) in heart disease diagnosis (33).

AI, especially deep learning and machine learning applications in the medical arena, is showing great potential in aiding clinicians and researchers to process and analyze large volumes of medical data. This is increasingly critical given the massive amounts of healthcare data being generated, and it's an area that has received significant endorsement from the research community.

### 4.3 Future trends

In recent times, there has been a notable increase in citations highlighting the potential of artificial intelligence (AI) in developing algorithmic models for predicting and diagnosing CVDs. This emerging area is anticipated to be a major research focus in the upcoming years. The application of AI in the realm of CVDs has gained significant traction, especially in the grading of cardiac arrhythmias and the diagnosis of coronary atherosclerotic heart disease.

#### 4.3.1 Artificial intelligence applications in cardiac arrhythmias

ECG has been widely used for decades as a commonly used tool in the diagnosis of cardiac arrhythmias. However, even if ECG recordings are well standardized and reproducible, artificial intelligence plays an important role in the exactness and efficiency of the diagnosis, as recognition of the images relies on the level of experience and expertise of the physician. While electrocardiography (ECG) has been a longstanding tool for diagnosing cardiac arrhythmias, the precision and efficiency of this process have been significantly enhanced by artificial intelligence (AI). Despite the well-standardized and reproducible nature of ECG recordings, the accuracy of diagnosis often depends on the experience and expertise of the interpreting physician.

In a 2020 study by Ribeiro et al., a dataset of 827 patients was collected and analyzed using a deep neural network (DNN) algorithm to discriminate and score ECGs. Notably, the study demonstrated high-performance measures for all ECG abnormalities, achieving F1 scores exceeding 80% and specificity indexes surpassing 99% (34). Siontis et al. (2021) further supported the efficacy of AI-enhanced ECG, highlighting its ability to detect subtle changes and identify undocumented CVDs, such as left ventricular dysfunction and hypertrophic cardiomyopathy (35). Moreover, the evolving use of CNNs, the predominant algorithm in deep learning, has significantly contributed to the classification of cardiac arrhythmias (36). These advancements in AI applications demonstrate a transformative impact on the accuracy and effectiveness of ECG-based cardiac diagnoses.

#### 4.3.2 Artificial intelligence in coronary heart disease

Noninvasive imaging, propelled by robust computational power, extensive datasets, and advanced models, stands as the cornerstone of contemporary cardiovascular diagnostics. Artificial intelligence (AI) is making significant strides in the field of carotid ultrasound measurements. Cardiovascular Magnetic Resonance Imaging (CMRI), a widely accepted tool for cardiovascular risk assessment, incorporates AI, particularly in image recognition, revolutionizing the diagnostic and prognostic analyses of cardiomyopathies using Late Gadolinium Enhancement (LGE) (37).

AI's role extends to identifying scar tissues (38) and minimizing artifacts in CMRI (39), thereby enhancing the speed and accuracy of diagnoses (30, 40). Notably, the development of three-dimensional convolutional neural networks has significantly improved the quantitative analysis of myocardial scar volume compared to traditional two-dimensional models (41). Studies, such as those led by Ali RL et al., exemplify the efficacy of assessing left atrium substrate using normalized intensity from LGE-CMRI (42), enabling risk stratification and personalized treatment strategies. However, it is crucial to acknowledge potential risks associated with contrast agents, such as contrast medium gadolinium (43).

In carotid ultrasound automated measurements play a pivotal role in boosting the efficiency and accuracy of carotid intima-media thickness (ciMT) and carotid intima-media thickness (PA) estimation, surpassing traditional manual methods. This AI-guided approach processes extensive data, including ciMT, PA (44), and Left Ventricle Ejection Fraction (LVEF) (45), thereby aiding clinical decision-making.

AI's ability to process massive datasets and conduct granular analysis marks a key development in cardiovascular diagnosis and patient care. Large language models like ChatGPT4 contribute to AI's role in decision-making by analyzing medical data and providing personalized cardiac treatment recommendations to identify potentially dangerous symptoms (46).

Despite the revolutionary potential of AI and machine learning in cardiovascular medicine, careful consideration must be given to potential risks and pitfalls. Ensuring proper training and validation of AI models is imperative to prevent inaccurate or misleading results. Ethical challenges (47), including responsibility ethics, doctor-patient ethics and digtal ethics (7), also demand attention. Balancing the transformative power of AI with patient safety is crucial in shaping the future of CVD prediction, diagnosis, and care.

The ongoing research trend revolves around leveraging AI in various aspects of CVD prediction, diagnosis, and treatment. AI's capability to handle extensive datasets and drive refined analyses presents a promising avenue for accurate and personalized treatments, representing a critical evolution in cardiovascular diagnostics and patient care. However, the integration of technologies like AI and machine learning must be approached with meticulous consideration for potential risks and pitfalls. The overarching goal is to harness AI's transformative capabilities while ensuring patient safety, thereby shaping the research landscape in CVD prediction and diagnosis.

### 4.4 Limitation

Our study is currently the only bibliometric analysis studying the application of AI in the CVDs field, but it has some limitations. The analysis was conducted in English only, which may introduce some language bias. Also, relying solely on citation analysis from WOSCC may inadvertently overlook some influential documents. Nonetheless, the WOSCC database contains a large amount of international literature and is currently the most commonly used database for bibliometric analysis. Despite language and database limitations, our comprehensive and up-to-date dataset for bibliometric and visual analysis provides a clear overview that will guide future research.

## 5 Conclusion

In conclusion, this research encompasses the first inclusive bibliometric analysis of publications concerning the role of AI in CVDs from 2000 to 2023. The findings indicate a substantial and expanding application of AI in CVDs. Although the United States currently leads in this field, significant growth is observed in China. It stresses the need for enhanced international cross-border collaborations, particularly involving Asian countries. Currently, cardiovascular imaging techniques and the selection of appropriate algorithms represent the most extensively studied areas, and a considerable boost in these areas is predicted in the coming years. Large language models as an emerging technology is causing huge vibrations in several fields, and it is no exception in the healthcare industry, which is also expected to see considerable growth in the field in the future (48).

## Data Availability

The original contributions presented in the study are included in the article/Supplementary Material, further inquiries can be directed to the corresponding author.
